# Three Quantitative Trait Loci Explain More than 60% of Variation for Chill Coma Recovery Time in a Natural Population of *Drosophila ananassae*

**DOI:** 10.1534/g3.119.400453

**Published:** 2019-09-19

**Authors:** Annabella Königer, Saad Arif, Sonja Grath

**Affiliations:** * Division of Evolutionary Biology, Ludwig-Maximilians-Universität (LMU) München, 82152 Planegg-Martinsried, Germany and; † Centre for Functional Genomics, Department of Biological and Medical Sciences, Oxford Brookes University, Oxford, OX3 0BP, United Kingdom

**Keywords:** QTL mapping, RAD-sequencing, cold tolerance, chill coma recovery time, *Drosophila ananassae*

## Abstract

Ectothermic species such as insects are particularly vulnerable to climatic fluctuations. Nevertheless, many insects that evolved and diversified in the tropics have successfully colonized temperate regions all over the globe. To shed light on the genetic basis of cold tolerance in such species, we conducted a quantitative trait locus (QTL) mapping experiment for chill coma recovery time (CCRT) in *Drosophila ananassae*, a cosmopolitan species that has expanded its range from tropical to temperate regions. We created a mapping population of recombinant inbred advanced intercross lines (RIAILs) from two founder strains with diverging CCRT phenotypes. The RIAILs were phenotyped for their CCRT and, together with the founder strains, genotyped for polymorphic markers with double-digest restriction site-associated DNA (ddRAD) sequencing. Using a hierarchical mapping approach that combined standard interval mapping and a multiple-QTL model, we mapped three QTL which altogether explained 64% of the phenotypic variance. For two of the identified QTL, we found evidence of epistasis. To narrow down the list of cold tolerance candidate genes, we cross-referenced the QTL intervals with genes that we previously identified as differentially expressed in response to cold in *D. ananassae*, and with thermotolerance candidate genes of *D. melanogaster*. Among the 58 differentially expressed genes that were contained within the QTL, *GF15058* showed a significant interaction of the CCRT phenotype and gene expression. Further, we identified the orthologs of four *D. melanogaster* thermotolerance candidate genes, *MtnA*, *klarsicht*, *CG5246* (*D.ana*/*GF17132*) and *CG10383* (*D.ana*/*GF14829*) as candidates for cold tolerance in *D. ananassae*.

Temperature is one of the major factors that influence the geographical distribution and abundance of ectothermic species. Physiological mechanisms to regulate body temperature are usually limited in ectotherms and resilience towards temperature extremes often determines the species fate upon climate change or range expansion. *Drosophila* spp. have successfully mastered such thermal challenges as they colonized temperate regions all over the globe and are now present on all of the earth’s continents except Antarctica (Lachaise *et al.*, 1988). By far the most prominent example of the genus is *Drosophila melanogaster*, which originated in sub-Saharan Africa, colonized temperate regions after the last glaciation about 15,000 years ago and nowadays has a worldwide distribution (David and Capy 1988; Stephan and Li 2007). In *D. melanogaster*, multiple candidate genes for cold tolerance have been identified (Sinclair *et al.*, 2007; Telonis-Scott *et al.*, 2009; Zhang *et al.*, 2011). Nevertheless, it remains challenging to link genotype and phenotype (von Heckel *et al.*, 2016; Udaka *et al.*, 2010) and it has been suggested that adaptation to local temperatures may have required simultaneous selection at multiple loci (Norry *et al.*, 2008; Tucić 1979).

Previously, we examined the cold tolerance of *Drosophila ananassae* (Königer and Grath 2018), a species of tropical origin that belongs to the Melanogaster species group (Clark *et al.*, 2007). The home range of *D. ananassae* was dated back to an expansion of the South-East-Asian continental shelf (’Sundaland’) during the late Pleistocene about 18,000 years ago (Das *et al.*, 2004). Since then, the species expanded to temperate regions and has nowadays a quasi-cosmopolitan distribution (Tobari 1993).

We measured cold tolerance by means of a test for chill coma recovery time (CCRT), which is defined as the time the flies need to stand on their legs after a cold-induced coma (David *et al.*, 1998). There was substantial variation in CCRT among fly strains that were derived from a population originating from the ancestral species range in Bangkok, Thailand. Most strikingly, there were two groups of strains within this population: cold-sensitive and cold-tolerant strains. While the cold-tolerant strains recovered as fast as strains from a temperate population (Kathmandu, Nepal) originating from the derived species range, the cold-sensitive strains had a significantly longer CCRT (Königer and Grath 2018). The difference between these two phenotypic groups was large if compared to within-population variation in *D. melanogaster* (von Heckel *et al.*, 2016). Thus, the phenotypes of the Bangkok strains could suggest that there is standing genetic variation for cold tolerance within this population. In a previous transcriptome analysis of the cold-sensitive and cold-tolerant strains from Bangkok, 15% of all protein-coding genes responded to the cold shock by either up- or downregulation. However, only two genes with unknown function, *GF15058* and *GF14647*, changed their transcript levels in a phenotype-specific manner after the cold shock, *i.e.*, their expression regulation differed significantly between the cold-tolerant and cold-sensitive phenotype (Königer and Grath 2018). *D. ananassae* displays high levels of population structure (Das *et al.*, 2004; Schug *et al.*, 2007) and the two distinct phenotypes indicate that there is genetic sub-structure within the Bangkok population as well. Further, the presence of genetic variation for cold tolerance in a tropical climate suggests pleiotropy that was maintained by balancing selection.

Hence, the present study was undertaken to gain better insight into the genetic architecture of cold tolerance in the Bangkok population of *D. ananassae*. We performed a genome-wide scan for quantitative trait loci (QTL) influencing CCRT in a mapping population of recombinant inbred advanced intercross lines (RIAILs) which was generated from the most cold-tolerant strain and the most cold-sensitive strain of the Bangkok population. By combining double-digest restriction site-associated DNA sequencing (ddRAD) markers and a hierarchical mapping approach, we identified three QTL of large effect which altogether explain 64% of the phenotypic variance in the mapping population. We further combined the present results with lists of genes that are differentially expressed in response to the cold shock in *D. ananassae* (Königer and Grath 2018) and *D. melanogaster* (von Heckel *et al.*, 2016).

Both species belong to the Melanogaster group and shared a common ancestor around 15-20 million years ago (Clark *et al.*, 2007). Our approach allowed us to narrow down the list of candidate genes for cold tolerance in *D. ananassae* and to point out potential commonalities between the two species.

## Materials and Methods

### Mapping population

**Figure 1:**
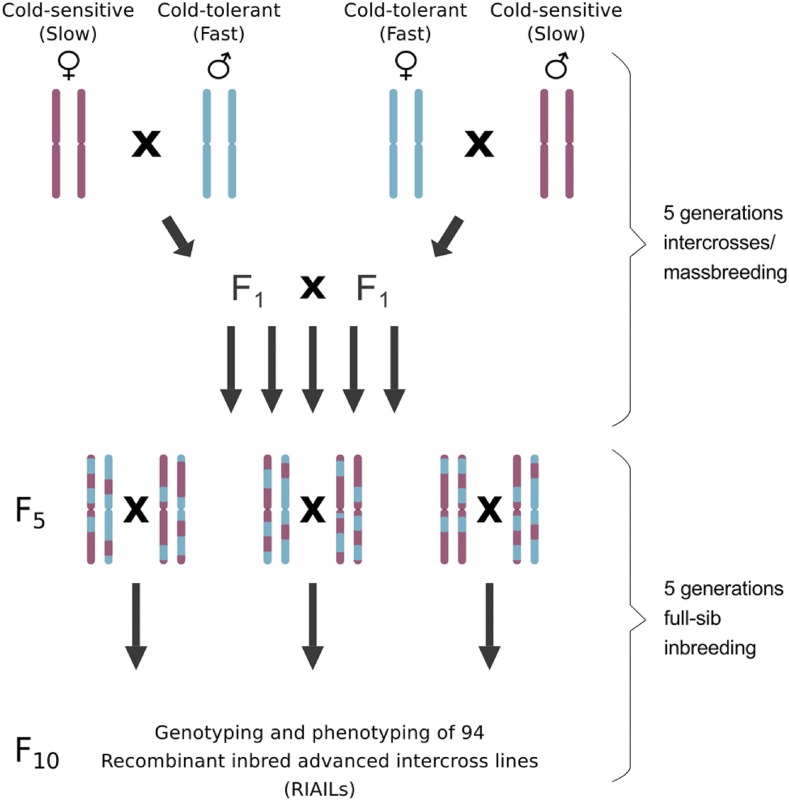
Crossing scheme for the generation of the RIAIL mapping population. Drawings of single chromosome pairs were used as representatives for the full genome. An initial, reciprocal cross between the cold-sensitive founder strain Slow (shown in red) and the cold-tolerant founder strain Fast (shown in blue) was set up to generate the heterozygous F1 generation. Intercrosses were continued in the form of massbreedings until generation F4, where single mating pairs were picked to allow for one more generation of intercrossing and to initiate inbreeding. From generation F5, full-sibling inbreeding was carried out for five subsequent generations. Throughout all generations (P - F10), the parents were removed before the offspring hatched to avoid back-crosses.

All flies used in this study were raised on standard cornmeal molasses medium, at constant room temperature (22 ± 1°) and at a 14:10 h light:dark cycle (details of the food recipe can be found in Königer and Grath 2018). The two fly strains (Fast and Slow) that were used as founders for the mapping population were collected in 2002 in Bangkok, Thailand, and established as isofemale strains (Das *et al.*, 2004). The strains were not selected for cold tolerance and maintained at low density at a population size of approximately 500 individuals. The phenotypic difference in chill coma recovery time (CCRT) among the Fast and Slow founder strains was maintained for at least eight years (see results section and Supplementary file 1: Table S1 and Table S2). Recombinant Inbred Advanced Intercross Lines (RIAILs) were generated as follows (Figure 1): two initial crosses between the two parental strains were set up (Fast males x Slow females and Slow males x Fast females). Individuals from both F1 generations were mixed and allowed to mate freely with each other. Up to generation F4, intercrossing was continued in the form of mass breedings. In generation F4, 360 mating pairs were set up in separate vials to allow for one more generation of intercrossing and to initiate the inbred strains. From generation F5, full-sibling inbreeding was carried out by mating brother-sister pairs for five subsequent generations. Throughout all generations (P - F10), the parents were removed before the offspring hatched to avoid back-crosses. From generation F10 on, RIAILs were kept at low density in 50 ml vials.

### Test for chill coma recovery time (CCRT)

CCRT was measured for flies of 4 – 6 days of age as described previously (Königer and Grath 2018). For the two founder strains Fast and Slow, CCRT was measured for males and females separately. For the RIAILs, only female flies were phenotyped. All female flies were collected and phenotyped as virgins. In brief, collection and sex-separation were carried out under light CO_2_-anesthesia, whereby ten flies from the same sex and strain were collected into a 50 ml vial containing 10 ml of cornmeal molasses medium. At the age of 4–6 days, the flies were transferred without anesthesia into new vials without food. For the cold shock, the vials were placed in an ice water bath (0 ± 0.5°) for exactly 3 h. Back at room temperature (22 ± 1°), CCRT was monitored in 2 min intervals for the duration of 90 min. Flies that were still not standing after 90 min were assigned a recovery time of 92 min. Flies that died during the experiment (< 1%) were excluded from the analysis. On average, we tested 40 female individuals per RIAIL and 100 individuals per founder strain and sex. In order to analyze the relationship between fly strain and CCRT in the two founder strains, we fitted a generalized linear mixed effects model on square root transformed measures for CCRT with lme4 (version 1.1-21) (Bates *et al.*, 2015) in R (version 3.6.1) (R Core Team 2018). Fly strain and sex were added as fixed effects and the intercept of each technical replicate was added as random effect to the model, while allowing for heterogeneous variances among the two fly lines. We then fitted two reduced models lacking either the sex or the line fixed effect. Significance of fixed effects was determined by likelihood ratio tests comparing the full model to each reduced model.

### DNA extraction and sequencing

DNA was extracted from 94 RIAILs and the two parental strains with the DNeasy Blood & Tissue Kit (QIAGEN, Hilden, Germany). For each strain, 10 virgin female individuals were pooled. DNA concentration and purity were assessed with a spectrophotometer (NanoDrop ND 1000, VWR International, Radnor, PA, USA). Library preparation and double-digest restriction site-associated DNA sequencing (ddRAD-seq) was carried out by an external sequencing service (ecogenics GmbH, Balgach, Switzerland) in the following way: DNA was double-digested with EcoRI and MseI and ligated to respective adapters comprising EcoRI and MseI restriction overhangs. Molecular identifier tags were added by polymerase chain reaction. The individual sample libraries were pooled, and the resulting library pools were size-selected for fragments between 500-600 bp with gel electrophoresis and extraction of the respective size range. The resulting size selected library pools were sequenced on a NextSeqTM 500 Sequencing System (Illumina, San Diego, CA), producing single-ended reads of 75bp length. Demultiplexing and trimming from Illumina adapter residuals was also carried out by the external service.

### Marker catalog construction and data curation

The software pipeline Stacks (version 1.45) (Catchen *et al.*, 2011) was used to analyze the sequence data and to identify markers. First, to examine the quality of the sequence reads, the *process_radtags* program was run in Stacks, applying a sliding window size of 50% of the read length (*-w 0.5*) to filter out reads which drop below a 99% probability of being correct (Phred score < 20) (*-s 20*). Second, the processed reads of each sample were mapped to the *D. ananassae* reference genome (FlyBase release 1.05 (Attrill *et al.*, 2016) with NextGenMap (version 0.5.0) (Sedlazeck *et al.*, 2013). Third, the mapped reads were converted to bam format, sorted and indexed with samtools (version 0.1.18) (Li *et al.*, 2009). Fourth, the *ref_map.pl* wrapper program was run in Stacks, which executes the Stacks core pipeline by running each of the Stacks components individually. In brief, *pstacks* assembled RAD loci for each sample, *cstacks* created a catalog of RAD loci from the two parental samples to create a set of all possible alleles expected in the mapping population and *sstacks* matched all RIAIL samples against the catalog. The *genotypes* program was executed last, applying automated corrections to the data (*-c*) to correct for false-negative heterozygote alleles. Only those loci which were present in at least 80% of the samples were exported (*-r 75*). Fifth, we applied additional corrections to the catalog by running the *rxstacks* program with the following filtering settings: non-biological haplotypes unlikely to occur in the population were pruned out (*–prune_haplo*), SNPs were recalled once sequencing errors were removed using the bounded SNP model (*–model_type_bounded*) with an error rate of 10% *(–bound_high 0.1*), and catalog loci with an average log likelihood less than –200 were removed (*–lnl_lim -200.00*). Sixth, *cstacks* and *sstacks* and *genotypes* (*-r 75*) were rerun to rebuild, match and export a new catalog with the filtered SNP calls. *Load_radtags.pl* and *index_radtags.pl* were used to upload and index the new catalog to a MySQL database. Seventh, a custom R script was used to remove markers with extreme values of residual heterozygosity within RIAILs, using cutoffs based on our inbreeding scheme (> 15% and < 35%) (Falconer and Mackay 1996) and to remove markers with an allele frequency drift < 10% from further analysis. Eighth, the MySQL database was used to manually check the markers for errors. Additionally, we investigated the proportion of residual heterozygosity within the two founder strains by counting the occurrence of heterozygous sites in the haplotype models. A total of 1,400 markers were included in the downstream analysis.

### Genetic map construction

Genetic map construction was conducted with R/qtl (version 1.42) (Broman *et al.*, 2003). The function *countXO* was used to remove seven RIAILs with > 200 crossover events. One more RIAIL was removed due to a low number of genotyped markers (< 700). The downstream analysis included 1,400 markers and 86 RIAIL-samples (Supplementary file 1: Table S4). Markers were partitioned into linkage groups based on a logarithm of the odds (LOD) score threshold of 8 and a maximum recombination frequency (rf) of 0.35, assuming a sequencing error rate of 1%. Map distances were calculated using the Haldane map function. As a sanity check, the functions *plotRF* and *checkAlleles* were used to test for potentially switched alleles and linkage groups were visually validated (based on rf and LOD scores).

### Analysis of quantitative trait loci (QTL)

QTL mapping was conducted with R/qtl (version 1.42) (Broman *et al.*, 2003). Prior to mapping, the genotype probabilities between marker positions were calculated with the function *calc.genoprob* on a maximum grid size of 1 cM. To identify major QTL, standard interval mapping was performed using the Expectation Maximization (EM) algorithm as implemented with the *scanone* function. The results are expressed as a LOD score (Sen and Churchill 2001). Significance thresholds were calculated with 1,000 genome-wide permutations. The initial single-QTL scan was extended with a more complex, two-dimensional scan using Haley-Knott-Regression as implemented with the *scantwo* function. Significance thresholds were again calculated with 1,000 genome-wide permutations.

To screen for additional QTL, estimate QTL effects and refine QTL positions, multiple-QTL mapping (MQM) was performed (Arends *et al.*, 2010). Here, missing genotypes were simulated from the joint distribution using a Hidden Markov model with 1,000 simulation replicates and an assumed error rate of 1% as implemented with the *sim.geno* function. The MQM model was identified with a forward selection/backward elimination search algorithm as implemented with the *stepwise* function, with the model choice criterion being penalized LOD scores. The penalties were derived on the basis of the significance permutations from the two-dimensional genome scan.

To estimate the support interval for each identified QTL, an approximate 95% Bayesian credible interval was calculated as implemented by the *bayesint* function. Gene annotations for QTL intervals were downloaded from FlyBase (Attrill *et al.*, 2016) and screened for enriched GO terms and KEGG pathways with DAVID (version 6.8) (Huang *et al.*, 2009). Enrichment was calculated against the background of all annotated genes (Attrill *et al.*, 2016) using default settings (EASE-score of 0.1 after multiple testing correction according to Benjamini-Hochberg (Benjamini and Hochberg 1995)).

In addition, we cross-referenced the QTL gene lists with lists of differentially expressed genes from a previously conducted transcriptome analysis, where we compared gene expression among cold-tolerant and cold-sensitive fly strains from the Bangkok population (including the two parental founder strains used in this study) in response to the 3 h cold shock at 0° (Königer and Grath 2018). Moreover, the transcriptome analysis also comprises lists of differentially expressed genes of cold-tolerant and cold-sensitive fly strains of *Drosophila melanogaster* in response to a cold shock (von Heckel *et al.*, 2016), allowing us to compare expression regulation of orthologous genes within the QTL regions among these two *Drosophila* species.

### Data Availability

File S1 contains phenotyping data, DNA concentrations and information on the QTL model. File S2 contains detailed information on all three identified QTL. Sequence data have been deposited in NCBI’s Sequence Read Archive and are accessible through series accession number PRJNA544044. Code used to perform the data analysis is available upon request. Supplemental material available at FigShare: https://doi.org/10.25387/g3.8288591.

## Results

### Chill coma recovery time (CCRT) Phenotype

**Figure 2:**
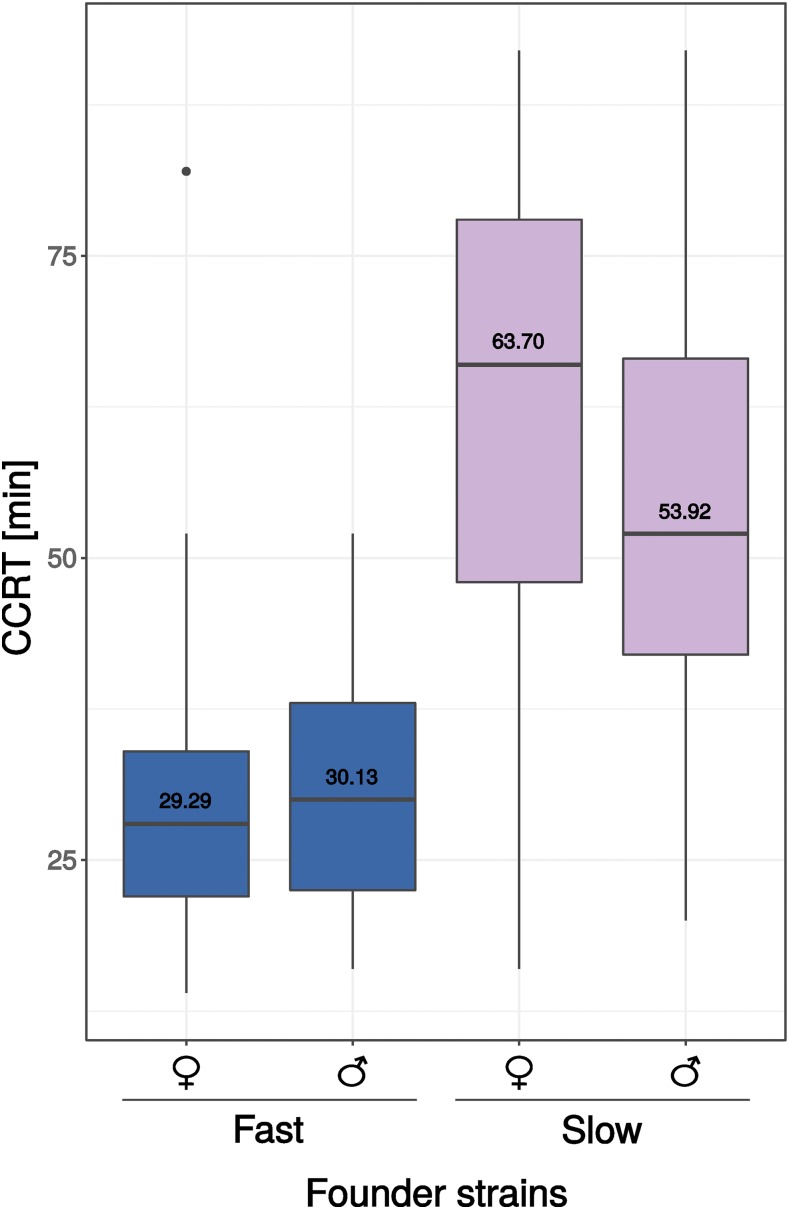
Chill coma recovery time (CCRT) of 4-6 day old flies of two strains of *D. ananassae* from Bangkok (Thailand) that were used as founder strains for the mapping population. The effect of the founder strain on CCRT was significant (Likelihood ratio test, *P*-value < 0.0001). The effect of sex on CCRT was not significant (Likelihood ratio test, *P*-value = 0.2522).

**Figure 3:**
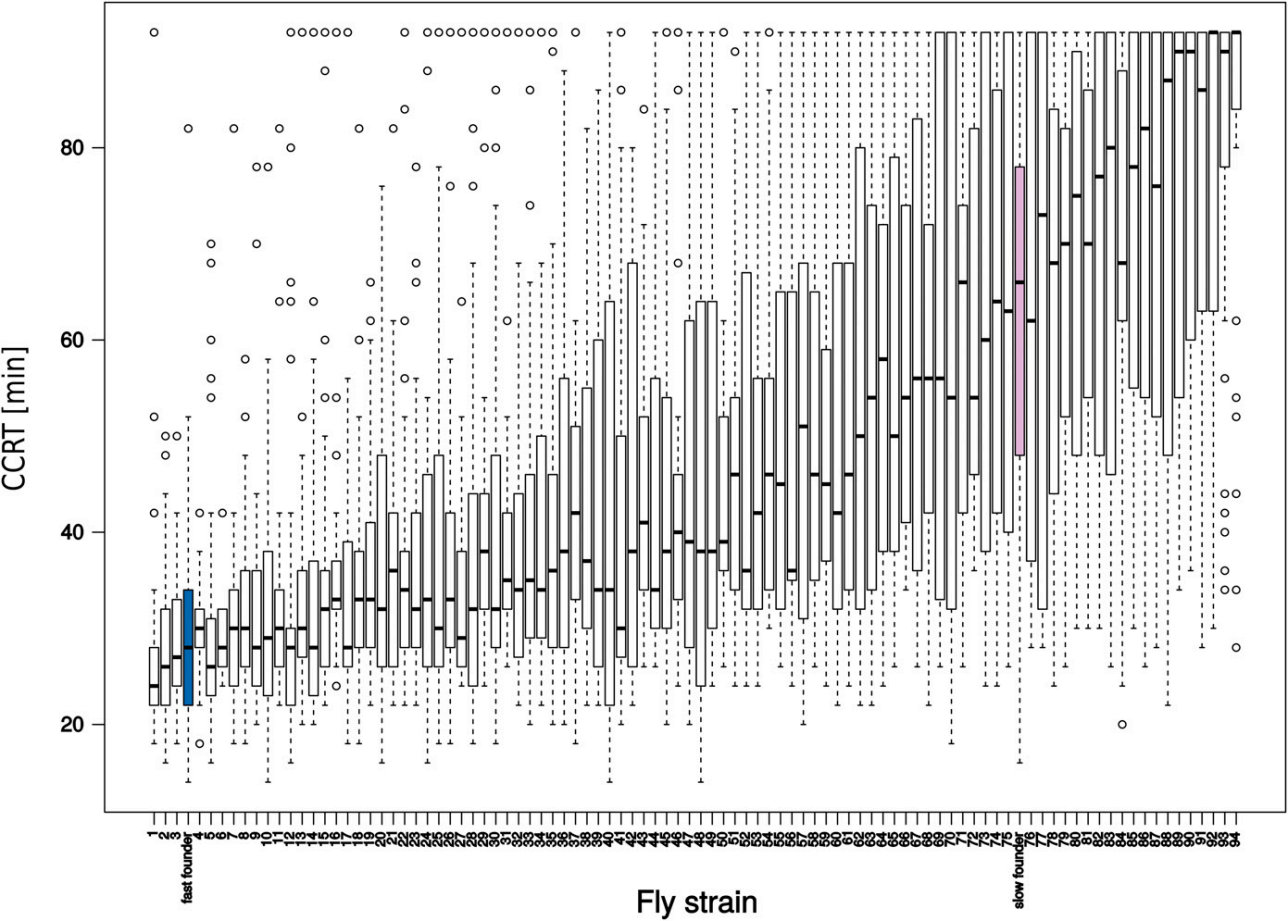
Chill coma recovery time (CCRT) of 4 - 6 day old virgin female flies of the 94 recombinant inbred advanced intercross lines (RIAILs) and the two founder strains. CCRT of the RIAILs is displayed with white bars. CCRT of the two founder strains is displayed in blue (Fast founder) and pink (Slow founder) (see also Figure 2). The RIAILs were numbered in ascending order according to their average CCRT. The fact that recovery times of 19 RIAILs exceeded the recovery time of the Slow founder strain suggests non-additive effects at causal loci.

The average CCRT of the cold-tolerant founder strain (Fast) was 29.29 min (standard deviation, SD, 9.90) for females and 30.13 min (SD 8.65) for males. CCRT of the cold-sensitive founder strain (Slow) was 63.70 min (SD 20.70) for females and 53.92 min (SD 18.40) for males (Figure 2, Supplementary file 1: Table S1). The effect of the founder strain on CCRT was significant (Likelihood ratio test, *P*-value < 0.0001). The effect of sex on CCRT was not significant (Likelihood ratio test, *P*-value = 0.2522) (see Supplementary file 1: Table S3). The average CCRT of the RIAILs ranged from 27.60 min to 83.03 min (Figure 3, Supplementary file 1: Table S4).

### Sequencing and genetic map

In total, we obtained 331,867,133 sequence reads with an average of 3,281,450 reads per sample. 0.6% of the total reads (2,074,057) failed the Stacks *process_radtags* quality check and were excluded from the analysis. In each of the samples, > 94% of all reads mapped to the *D. ananassae* reference genome. The amount of residual heterozygosity within the founder strain sequences was <0.5% (Supplementary file 1: Table S9). The Stacks core pipeline matched 5,468 markers to the initial catalog. After additional corrections with the *rxstacks* program, 3,092 markers remained. From this new catalog, 691 markers were excluded due to extreme values of heterozygosity (>35% or < 15%), 735 markers were excluded due to high levels of allele frequency drift (> 10%) and 266 markers were excluded that fell into both categories. Thus, after all filtering steps, a total of 1,400 markers and 86 RIAILs were used for genetic map construction. The markers were partitioned into eight linkage groups (Supplementary file 1: Table S6). The total map length was 962.0 cM, with an average marker spacing of 0.7 cM and a maximum marker spacing of 55.5 cM (Figure 5). Across all samples, 91.6% of the genotypes were available of which 37.4% were homozygous for the cold-tolerant (Fast) allele (FF), 27.9% were heterozygous (FS) and 34.7% were homozygous for the cold-sensitive (Slow) allele (SS).

### One- and two-dimensional genome scans

**Figure 4:**
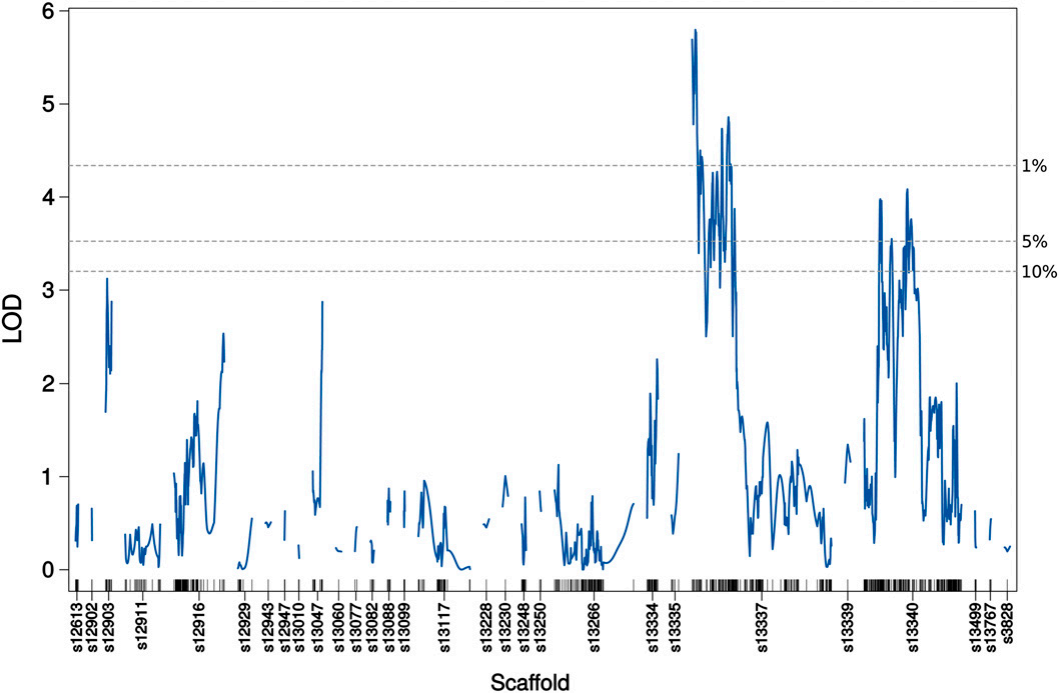
LOD-curves obtained with standard interval mapping reveal two significant QTL, QTL1 on scaffold 13337 and QTL2 on scaffold 13340. Significance thresholds (dotted lines) were calculated with 1,000 genome-wide permutations. The short vertical lines on the X-axis correspond to the marker positions.

Interval mapping in the context of a single-QTL model revealed two major areas with LOD peaks which exceeded the permuted 5% significance level (LOD 3.53), one on scaffold 13337 (QTL1) and one on scaffold 13340 (QTL2) (Figure 4). The highest peak on scaffold 13337 was at 6.08 cM (LOD 5.80) and the highest peak on scaffold 13340 was at 80.05 cM (LOD 4.08).

The next step was to extend the initial, single-QTL scan with a two-dimensional scan, where we compared two possible models: the full (epistatic) model (H_f1_) which allowed for the possibility of a second QTL and interactions among QTL was compared to the additive model (H_a1_) which allowed for the possibility of a second QTL without interaction. Both the full and the additive model reached maximum LOD scores at the same positions, 7.08 cM on scaffold 13337 and 30.1 cM on scaffold 13340 (Table 1). In comparison to the single-QTL model, we found supporting evidence for the presence of a second QTL under the additive model (lodd.av1 *P*-value = 0.006), but not under the full model (lod.fv1 *P*-value = 0.668). There is no evidence for interaction among the two loci (lod.int *P*-value = 1).

**Table 1:** Results of the two-dimensional genome scan

Two-QTL scan								
	pos1f*^a^*	pos2f*^a^*	lod.full*^a^*	*P*-value*^a^*	lod.fv1*^b^*	*P*-value*^b^*		
s13337:s13340	7.08	30.1	12.6	0	5.77	0.668		
	pos1f*^c^*	pos2a*^c^*	lod.add*^c^*	*P*-value*^c^*	lod.av1*^d^*	*P*-value*^d^*	lod.int*^e^*	*P*-value*^e^*
s13337:s13340	7.08	30.1	11.4	0	4.54	0.006	1.24	1

a QTL positions, LOD score and *P*-value for the full (epistatic) model *vs.* the Null-model.

b LOD score and *P*-value for the full (epistatic) model *vs.* the Single-QTL-model.

c QTL positions, LOD score and *P*-value for the additive model *vs.* the Null-model.

d LOD score and *P*-value for the additive model *vs.* the Single-QTL-model.

e LOD-score and *P*-value of (full model – additive model) = evidence for interaction.

*P*-values represent the proportion of permutation replicates with LOD scores ≥ the observed.

### Multilpe-QTL model

**Figure 5:**
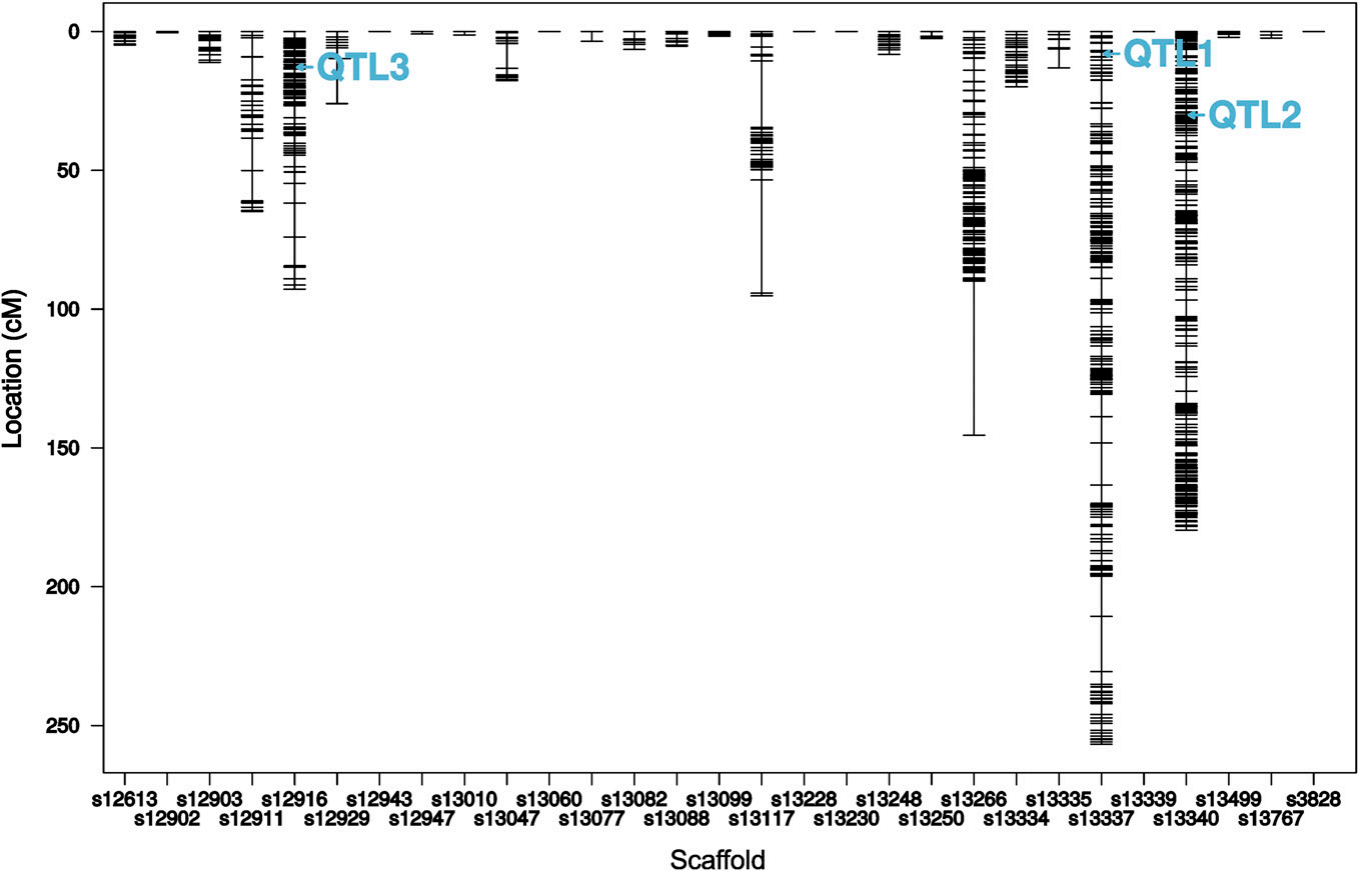
Genetic map with QTL positions as refined with the multiple-QTL model. X-axis = genomic scaffolds. Y-axis = genetic distances in centiMorgan (cM) for markers (short horizontal lines).

**Figure 6:**
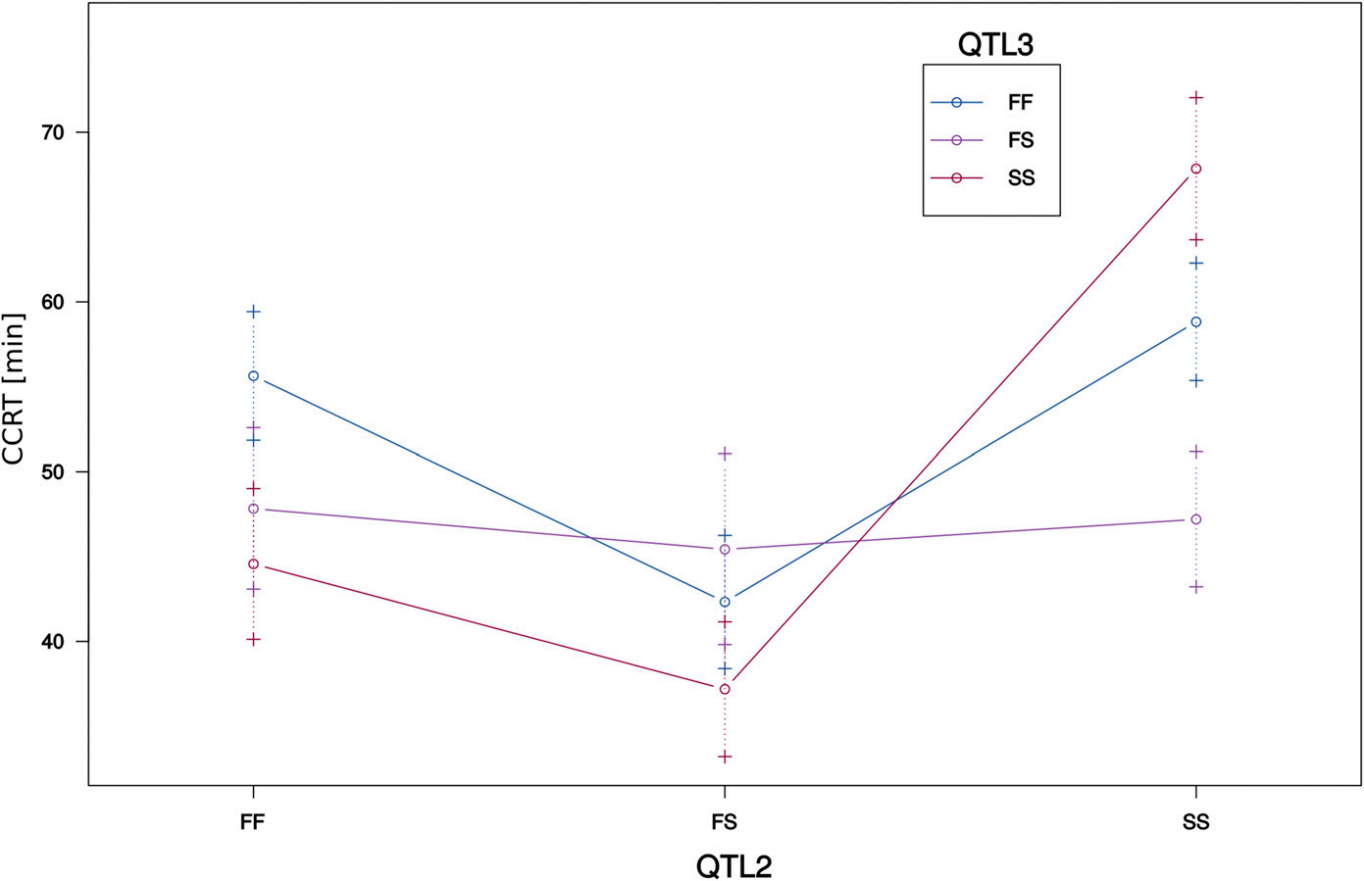
Interaction of QTL2 on scaffold 13340 and QTL3 on scaffold 12916. X-axis = genotypes for QTL2. The genotypes for QTL3 are represented by lines in different colors. Error bars are plotted at +/− 1 SE. F = cold-tolerant parental allele (fast CCR), S = cold-sensitive parental allele (slow CCR).

In order to identify possible additional QTL of moderate effect, refine QTL positions, separate linked loci and to estimate QTL effects, we applied a forward selection/backward elimination algorithm with penalized LOD scores and identified a model with three main terms and one interaction term. The overall fit of the model had a LOD score of 19.26 and explained 64.34% of the phenotypic variance (Figure S2, Supplementary file 1: Table S7). In comparison to the one- and two-dimensional genome-scans, there was an additional locus on scaffold 12916 at position 16.7 cM (QTL3) which interacted with one of the previously identified loci, on scaffold 13340 (QTL2) (Table 2, Figures 5, 6 and Figure S3).

**Table 2:** QTL confidence intervals and estimated effects

	Scaffold	cytologic position [cM]	cytologic position [bp]	confidence interval [bp]	% variance	additive effect	dominance deviation	genes*^a^*
QTL1	13337	0.083871	0.083871 – 9.233870	83.871 – 226.785	26.59	9.2082	−2.1719	11
QTL2	13340	30.053110	27.51427 – 36.52024	5.544.039 – 6.544.039	30.44	1.7317	−5.2343	138
QTL3	12916	16.747634	7.103214 – 92.933043	1.514.827 – 2.696.582	19.89	−0.6307	−0.8054	110

QTL positions and effects on the phenotype as estimated with the multiple-QTL model. Confidence intervals were calculated as 95% Bayesian credible intervals.

a Numbers of protein-coding genes within QTL intervals. Numbers and identifiers for non-coding genes and RNAs are shown in Supplementary file 2.

QTL effects were estimated for additivity ((SS-FF)/2) and deviation from dominance ((FS-(FF+SS)/2), where F denotes the cold-tolerant Fast allele and S denotes the cold-sensitive Slow allele (Table 2, Supplementary file 1: Table S8). QTL3 on scaffold 12916 was a transgressive QTL, as the cold-tolerant allele was associated with having a more cold-sensitive phenotype (longer CCRT), resulting in a negative effect size (Figure S1C). For QTL1, the estimated additive effect was positive while the estimated dominance effect was negative. RIAILs homozygous for the cold-tolerant allele had the most cold-tolerant phenotype, RIAILs homozygous for the cold-sensitive allele had the least cold-tolerant phenotype and heterozygote RIAILs had an intermediate phenotype (Figure S1A). The effect estimates for QTL2 went in the same direction as for QTL1. Here, however, the heterozygous phenotype was associated with the most cold-tolerant phenotype (Figure S1B). The more complex relationships of additive and dominance effects for the interaction of QTL3 and QTL2 can be understood best by plotting the interaction of the phenotype and the genotype at both marker positions (Figure 6).

As revealed by the interaction plot (Figure 6), RIAILs homozygous for the cold-sensitive (S) allele at both QTL also had the most cold-sensitive phenotype. The most cold-tolerant phenotype, was reached by those RIAILs which were homozygous for the cold-tolerant allele at QTL2 but homozygous for the cold-sensitive allele at (the transgressive) QTL3. Interestingly, cold tolerance of RIAILs which were heterozygous at QTL3 seemed to be independent from their genotype at QTL2.

The results of a drop-one-term at a time ANOVA indicated strong evidence for all three loci and the interaction of QTL2 and QTL3. For each QTL, the model with the QTL of interest at that particular position was compared to the model with the QTL of interest omitted, while all other QTL positions were fixed at their maximum likelihood estimates (Table 3, Figure S3).

**Table 3:** Summary table for the drop one term ANOVA

QTL	cytologic position	df	Type III SS	LOD	% Var	F value	*P* (Chi^2^)	*P* (F)
1	S13337-0.1	2	4903	10.404	26.59	27.962	0	8.45E-10
2	S13340-30.1	6	5613	11.526	30.44	10.671	0	1.52E-08
3	S12916-16.7	6	3667	8.277	19.89	6.972	0	6.47E-06
2:3	S13340-30.1:s12916-16.7	4	2790	6.606	15.13	7.957	0	2.11E-05

S13337-0.1 = QTL on scaffold 13337 at position 0.1 cM, df = degrees of freedom, SS = sums of squares, MS = mean squares, LOD = relative to the null model, %Var = proportion of variance in the phenotype explained by all terms in the model, *P* (Chi2) = *P*-value based on LOD score following a χ 2-distribution, *P*(F) = *P*-value based on the F-statistic. Profile LOD scores are shown in Figure S3.

### Candidate gene meta analysis

All three QTL together contained 259 protein-coding genes (Table 2, Supplementary file 2: Tables S1, S2, S5). Among them were 58 differentially expressed genes (Table 4), which was significantly more than we would expect by chance (Fisher’s exact test, *P* = 0.02867).

**Table 4:** Cold tolerance candidate genes within QTL regions

	DE genes	Cold tolerance candidate genes
QTL1	3	*GF24896* (*D.mel*/*klarsicht*, MacMillan *et al.*, 2016)
QTL2	26	*MtnA* (*D.mel*/*MtnA*, Catalán *et al.*, 2016), *GF17132* (*D.mel*/*CG5246*, von Heckel *et al.*, 2016)
QTL3	29	*GF14829* (*D.mel*/*CG10383*, Norry *et al.*, 2008), *GF15058* (Königer and Grath 2018)

DE genes = differentially expressed genes in response to the cold shock as identified by Königer and Grath, 2018. DE genes are listed in Supplementary file 2: Tables S1, S2 and S5.

Cold tolerance candidate genes = genes previously identified as candidates for cold tolerance in *D. melanogaster.*

QTL1 spanned 140 kb and contained eleven protein coding genes (Supplementary file 2: Table S1). There was no enrichment of KEGG pathways or GO terms. However, three of the eleven genes were previously identified to be differentially expressed in response to a cold shock (Supplementary file 2: Table S1, (Königer and Grath 2018). Two of them, *GF24884* (ortholog of *p130CAS*) and *GF24880* (ortholog of *Phosphoinositide-dependent kinase 1*), were upregulated in both phenotypes after the cold shock and one of them, *GF24896* (ortholog of *klarsicht*), was exclusively upregulated in the cold-tolerant phenotype only. Klarsicht was previously reported as upregulated in cold-acclimated flies of *D. melanogaster* (MacMillan *et al.*, 2016).

QTL2 spanned 1.0 Mb and contained 138 protein-coding genes which were enriched in one molecular function, “serine-type endopeptidase activity” (GO:0004252) and one biological process, “intracellular cholesterol transport” (GO:0032367) (Supplementary file 2: Table S3). Out of the 138 genes, 26 were previously identified as differentially expressed in response to a cold shock. Among them, nine genes were upregulated and five genes were downregulated in both phenotypes (see Supplementary file 2: Table S2, and (Königer and Grath 2018)). In the cold-tolerant phenotype, one gene, *GF17809* (ortholog of *Archease*), was exclusively upregulated and one gene, *GF17856* (ortholog of *Niemann-Pick type C-2c*), was exclusively downregulated. In the cold-sensitive phenotype, one gene, *GF17176* (ortholog of *aluminum tubes*), was exclusively upregulated and nine genes were exclusively downregulated (see Supplementary file 2: Table S2, and (Königer and Grath 2018).

**Figure 7:**
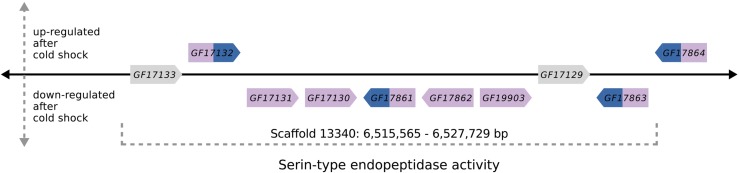
Schematic illustration of a genomic region within QTL2 that contains nine genes of the enriched GO category “serine-type endopeptidase activity” (see also Supplementary file 2: Table S2 and S3). Genes were differentially expressed in response to the cold shock in either the cold-sensitive (slow) phenotype alone (genes shown in pink color) or in both phenotypes, cold-sensitive and cold-tolerant (fast) (genes shown in blue and pink color). Genes that were not differentially expressed are shown in gray. Gene lengths and distances between genes are not drawn to scale.

Nine genes drove the enrichment in the GO category “serine-type endopeptidase activity” (see Supplementary file 2: Table S3). All of them were located in the downstream region of QTL2 at 6,515,565 - 6,527,729 bp and adjacent to one another (Figure 7). Seven of these genes were also differentially expressed in response to cold shock. Among them was *GF17132*, which was upregulated in both phenotypes and its ortholog in *D. melanogaster* showed a significant interaction of phenotype and cold shock (Supplementary file 2: Table S2, von Heckel *et al.*, 2016). QTL2 also contained the gene *Metallothionein A* (*MtnA*) which caught our attention because it is involved in metal ion homeostasis and in its *D. melanogaster* ortholog, an InDel polymorphism is associated with local adaptation to oxidative stress upon migration out of Sub-Saharan Africa into Europe (Catalán *et al.*, 2016). *MtnA* was downregulated in response to cold in *D. melanogaster* (von Heckel *et al.*, 2016) but not in *D. ananassae* (Königer and Grath 2018).

QTL3 spanned 1.2 Mb and contained 110 protein coding genes which were enriched in three molecular functions: “sequence-specific DNA binding” (GO:0043565), “ATPase activity” (GO:0016887) and “phosphotransferase activity, alcohol group as acceptor” (GO:0016773) and one KEGG pathway: “Hippo signaling pathway – fly” (dan04391) (Supplementary file 2: Table S6). Out of the 110 genes, 29 were previously identified as differentially expressed in response to a cold shock (Table 4). Among them, 12 genes were upregulated and seven genes were downregulated in both phenotypic groups, cold-tolerant and cold-sensitive (see Supplementary file 2: Table S5, (Königer and Grath 2018)). In the cold-tolerant phenotype, two genes, *GF15043* (ortholog of *CG31974*) and *GF14846* (ortholog of *bicoid** stability factor*), were exclusively upregulated and two genes, *GF15020* (ortholog of *ABC transporter expressed in trachea*) and *GF14865* (ortholog of *CG11454*), were exclusively downregulated. In the cold-sensitive phenotype, five genes were exclusively downregulated but there were no exclusively upregulated genes. One of the five downregulated genes was *GF15058* (ortholog of *CG10178*), which was one out of two genes with a significant interaction of phenotype and cold shock. The function of *GF15058* is unknown but it is predicted to have UDP-glycosyl-transferase-activity (Marchler-Bauer *et al.*, 2015).

## Discussion

We used a panel of 86 recombinant inbred advanced intercross lines (RIAILs) and 1,400 ddRAD markers to map QTL that underlie natural variation in cold tolerance among two fly strains of *D. ananassae* from a population in Bangkok, Thailand. The recovery time segregated significantly between the two founder strains. CCRT in the cold-sensitive strain was about twice as high as in the cold-tolerant strain. The three identified QTL for CCRT explain as much as 64% of the variance in the phenotype. This proportion is equal to a previous mapping experiment for CCRT in *D. melanogaster*, in which three QTL explained 64% of the variance for CCRT in an intercontinental set of recombinant inbred lines (Norry *et al.*, 2008). The founder strains for this mapping population were sampled from Denmark and Australia and thus from two geographically different thermal environments. Another study (Morgan and Mackay 2006) also identified three QTL for CCRT in *D. melanogaster* in a set of recombinant inbred lines derived from two laboratory strains that differed significantly for the phenotype. In this mapping population, the three loci explained 25% of the phenotypic variance for CCRT. While two of the reported QTL for CCRT in *D. melanogaster* co-localized across these two studies, none of the reported candidate genes co-localize with the QTL intervals in *D. ananassae* (this study).

Compared to sequencing of pooled samples (Pool-sequencing), RAD-based approaches come at the cost of marker density, especially in crossing designs with low genetic differentiation between the founder strains and low levels of linkage disequilibrium (Futschik and Schlötterer 2010). Thus, to increase the mapping resolution and to expand the genetic map, we generated a mapping population in which five generations of intercrosses allowed for a sufficient number of crossover events (Pollard 2012). Subsequently, we used stringent filtering cutoffs to account for potential sequencing errors. We thereby excluded a relatively high number of markers which did not meet our criteria for expected levels of hererozygosity and allele frequency drift. While this step certainly increased the robustness of the identified loci, estimates for deviations from dominance may be biased by segregation distortion or inbreeding effects. Eventually, excluding these markers came at the cost of chromosomal coverage, as many smaller genomic scaffolds were excluded from the analysis at this step. It is therefore possible that our results do not cover all potential QTL. However, the reduction of genome complexity that results from RAD-sequencing has two major benefits. First, it is more cost-effective than whole-genome sequencing of individual samples, allowing for a larger number of samples to be analyzed and consequently for greater statistical power to detect QTL. Second, it is more accurate than whole-genome Pool-sequencing (Catchen *et al.*, 2017; Cutler and Jensen 2010).

Populations of *D. ananassae* segregate for several polymorphic inversions, the frequencies of which follow latitudinal clines (Singh and Singh 2007). Moreover, chromosomal inversions appear to be temperature-sensitive in *D. ananassae* (Dasmohapatra *et al.*, 1982), which has also been demonstrated for *D. melanogaster* (Norry *et al.*, 2008). A cold tolerance factor that is located within an inversion could rise quickly in frequency upon seasonal fluctuations in temperature or upon local adaptation to temperate habitats by suppressed recombination in heterozygous karyotypes (Kirkpatrick and Barton 2006) - and thus confound QTL mapping. However, the CCRT phenotypes of the mapping population were distributed on a continuum (Figure 3), which speaks against the influence of a major inversion. Furthermore, there was no apparent excess of residual heterozygosity within the three QTL scaffolds in the founder genomes. Generally, detecting inversions from short-read ddRAD-sequences only is difficult. Therefore, we can neither confirm nor deny the presence of inversions in the tested strains.

Additionally, it needs to be noted that, in general, QTL confidence intervals should be considered as support regions rather than absolute boundaries (Broman and Sen 2009) and the causal genetic variants may be located anywhere within these intervals. However, combining the identified intervals with a previous transcriptome analysis in *D. ananassae* (Königer and Grath 2018) allowed us to narrow down the list of potentially causal genes.

The identification of candidate genes for cold tolerance has been extensively addressed in *D. melanogaster*. Like *D. ananassae*, *D. melanogaster* is of tropical origin (David *et al.*, 2007), but its thermal range (12-32°) (Moreteau *et al.*, 1997) is broader than the thermal range of *D. ananassae* (16-32°) (Morin *et al.*, 1997). Since both species adapted independently to temperate regions, it is unclear if the same genes that underly cold tolerance in *D. melanogaster* are also of importance in *D. ananassae*. Hence, we screened the QTL intervals in *D. ananassae* for cold tolerance candidate genes in *D. melanogaster* (von Heckel *et al.*, 2016; MacMillan *et al.*, 2016; Norry *et al.*, 2008; Ramnarine *et al.*, 2019) to highlight commonalities and differences among the two species.

From the combined data, we identified three types of candidates:

- The expression profile of *GF15058* (*D.mel*/*CG10178*) in QTL3 is directly associated with a difference in the CCRT phenotype in *D. ananassae*. *GF15058* was one out of two genes that responded to the cold shock in a phenotype-specific way (Königer and Grath 2018). Its function was inferred from electronic annotation to be uridine diphosphate (UDP) glycosyltransferase activity. UDP-glycosyltransferases (UGTs) are membrane-bound enzymes that are located in the endoplasmatic reticulum and catalyze the addition of a glycosyl group from a uridine triphosphate (UTP) sugar to a small hydrophobic molecule. Therefore, UGTs play an essential role in maintaining homeostatic function and detoxification and are known as major members of phase II drug metabolizing enzymes (Bock 2016). The cold shock led to a downregulation of *GF15058* in the Slow strains but not in the Fast strains. However, the Fast genotype at QTL3 is transgressive, *i.e.*, it increases CCRT. Thus, if *GF15058* was indeed one of the causal factors, our results suggest that keeping transcript abundance at a constant level after the cold shock is so costly for the organism that it slows down recovery.
- The expression profile of *GF17132* (*D.mel*/*CG5246*) in QTL2 is directly associated with a difference in the CCRT phenotype in *D. melanogaster*, where it showed a significant interaction of phenotype and cold shock (von Heckel *et al.*, 2016). It was also differentially expressed in response to the cold shock in *D. ananassae* (Königer and Grath 2018). Moreover, *GF17132* belongs to a cluster of genes that code for serine peptidases in QTL2 (Figure 7). Serine peptidases are involved in proteolysis, *i.e.*, they catalyze the hydrolysis of peptide bonds (Attrill *et al.*, 2016; Ross *et al.*, 2003). This process plays a central role in the immune response of insects (De Gregorio *et al.*, 2001) and serine proteases were suggested previously to be involved in the cold stress response as well (Vermeulen *et al.*, 2013).
- We identified three more genes that have been associated with thermotolerance in experiments other than the transcriptome analyses: *MtnA* (*D.mel*/*MtnA*), *GF24896* (*D.mel/klarsicht)* and *GF14829* (*D.mel/**CG10383*).

The gene *MtnA* in QTL2 codes for metallothionein A which promotes resistance to oxidative stress. It binds heavy metals and neutralizes reactive oxygen and nitrogen species (Ruttkay-Nedecky *et al.*, 2013). Exposure to cold leads to an increased abundance of free radicals, thereby inducing oxidative stress (Williams *et al.*, 2014). In *D. melanogaster*, a 49 bp deletion in the 3′UTR of *MtnA* is associated with its transcriptional upregulation and with increased tolerance to oxidative stress (Catalán *et al.*, 2016). The frequency of this polymorphism in natural populations follows latitudinal clines, suggesting that upregulation of *MtnA* is favored in temperate environments (Ramnarine *et al.*, 2019). However, a direct link between cold stress and oxidative stress is yet to be established in drosophilids (Plantamp *et al.*, 2016). *MtnA* was downregulated after the cold shock in both phenotypes of *D. melanogaster* (von Heckel *et al.*, 2016) and not differentially expressed in *D. ananassae*. Moreover, a previous sequence analysis of *MtnA* in *D. ananassae* reported the 3′UTR deletion polymorphism as absent in 110 strains that were sampled in tropical and temperate regions around the world (Stephan *et al.*, 1994).

The gene *GF24896* (*D.mel/**klarsicht*) in QTL1 is expressed in a wide range of tissues, where it interacts with microtubules and promotes evenly spaced positioning of nuclei. Knock-out of *klarsicht* in muscle cells impairs locomotion and flight (Elhanany-Tamir *et al.*, 2012) – functions that are also disabled during chill coma. The gene was reported previously to be upregulated with cold-acclimation in *D. melanogaster* (MacMillan *et al.*, 2016). In *D. ananassae*, *GF24896* is upregulated after the cold shock in fast strains but not in slow strains (Königer and Grath 2018), suggesting a potential contribution of this gene to faster recovery from cold exposure.

Lastly, the gene *GF14829* (*D.mel*/*CG10383*) in QTL3 is involved in the regulation of glycosylphosphatidylinositol metabolism. After the cold shock, it is upregulated in fast and slow strains of *D. ananassae* and in Slow strains of *D. melanogaster*. Interestingly, over-expression of *CG10383* increases lifespan in *D. melanogaster* (Paik *et al.*, 2012). It was also the only gene within all three QTL for CCRT in *D. ananassae* that mapped to a heat-tolerance QTL in *D. melanogaster* (Norry *et al.*, 2008). In the face of the transgressive nature of QTL3, potential allelic effects resulting in trade-offs between CCRT, heat-resistance and lifespan should be investigated in both species.

In conclusion, we identified three large-effect QTL for recovery from cold exposure in *D. ananassae*. Combining the present results with previous results obtained from *D. melanogaster* allowed us to shed light on commonalities and differences in the genetic basis of cold tolerance between these two species. The combined data point at the five above mentioned genes as candidates for recovery from cold exposure. Building on the present results, re-sequencing of the QTL intervals in cold-tolerant and cold-sensitive strains from Bangkok and additional strains from temperate populations may uncover signatures of selection. Moreover, the identified genes serve as the groundwork for more detailed analyses such as loss-of-function experiments to establish a link between genotype and phenotype in both species.
